# Disulfidptosis features and prognosis in head and neck squamous cell carcinoma patients: unveiling and validating the prognostic signature across cohorts

**DOI:** 10.1007/s00432-024-05691-9

**Published:** 2024-03-25

**Authors:** Hao Xue, Qianyu Sun, Heqing Zhang, Hanxiao Huang, Haowei Xue

**Affiliations:** Department of Stomatology, The First Affiliated Hospital of Anhui Medical University, Hefei, 230022 Anhui China

**Keywords:** Head and neck squamous cell carcinoma, Disulfidptosis, Prognosis, Nomogram

## Abstract

**Background:**

Head and neck squamous cell carcinoma (HNSCC) is a significant health concern with a variable global incidence and is linked to regional lifestyle factors and HPV infections. Despite treatment advances, patient prognosis remains variable, necessitating an understanding of its molecular mechanisms and the identification of reliable prognostic biomarkers.

**Methods:**

We analyzed 959 HNSCC samples and employed batch correction to obtain consistent transcriptomic data across cohorts. We examined 79 disulfidptosis-related genes to determine consensus clusters and utilized high-throughput sequencing to identify genetic heterogeneity within tumors. We established a disulfidptosis prognostic signature (DSPS) using least absolute shrinkage and selection operator (LASSO) regression and developed a prognostic nomogram integrating the DSPS with clinical factors. Personalized chemotherapy prediction was performed using the "pRRophetic" R package.

**Results:**

Batch corrections were used to harmonize gene expression data, revealing two distinct disulfidptosis subtypes, C1 and C2, with differential gene expression and survival outcomes. Subtype C1, characterized by increased expression of the MYH family genes ACTB, ACTN2, and FLNC, had a mortality rate of 48.4%, while subtype C2 had a mortality rate of 38.7% (HR = 0.77, 95% CI: 0.633–0.934, *P* = 0.008). LASSO regression identified 15 genes that composed the DSPS prognostic model, which independently predicted survival (HR = 2.055, 95% CI: 1.420–2.975, *P* < 0.001). The prognostic nomogram, which included the DSPS, age, and tumor stage, predicted survival with AUC values of 0.686, 0.704, and 0.789 at 3, 5, and 8 years, respectively, indicating strong predictive capability. In the external validation cohort (cohort B), the DSPS successfully identified patients at greater risk, with worse overall survival outcomes in the high-DSPS subgroup (HR = 1.54, 95% CI: 1.17–2.023, *P* = 0.002) and AUC values of 0.601, 0.644, 0.636, and 0.748 at 3, 5, 8, and 10 years, respectively, confirming the model's robustness.

**Conclusion:**

The DSPS provides a robust prognostic tool for HNSCC, underscoring the complexity of this disease and the potential for tailored treatment strategies. This study highlights the importance of molecular signatures in oncology, offering a step toward personalized medicine and improved patient outcomes in HNSCC management.

## Introduction

Head and neck squamous cell carcinoma (HNSCC) is recognized as a significant global public health concern, representing a diverse group of cancers that affect various regions, including the oral cavity, pharynx, hypopharynx, larynx, nasal cavity, and salivary glands, and is characterized by high incidence and mortality rates. The incidence of HNSCC varies worldwide and is influenced by regional factors such as tobacco and alcohol use, dietary habits, and the prevalence of human papillomavirus (HPV) infections (Barsouk et al. 2023; Kumar et al. 2015; Auguste et al. 2020). The elevated incidence of HNSCC in Southeast Asia and Australia correlates with the intake of particular products containing carcinogens. Concurrently, the increasing incidence of oropharyngeal infections due to HPV has been linked to the increased incidence of HNSCC in the USA and Western Europe (Mehanna et al. 2013; Kanwal et al. 2019; Su et al. 2016).

Despite advancements in diagnosis and treatment, the prognosis for HNSCC patients remains variable and often depends on the stage at diagnosis, the tumor's location, and the patient's overall health. HNSCC constitutes approximately 95% of all head and neck cancers, contributes to more than 316,000 deaths annually worldwide (Burden et al. 1990), and is the sixth most prevalent malignancy (Mody et al. 2021). Predictive analyses indicate a projected 30% increase in the risk of HNSCC by the year 2030, with an anticipated 1.08 million new cases annually (Antra 2022). Moreover, survival rates for HNSCC patients have improved moderately over the last 30 years; for instance, the 5-year survival rate increased from 55% in 1992–1996 to 66% in 2002–2006, as per the Surveillance, Epidemiology, and End Results registry data encompassing all age groups and anatomical locations (Pulte and Brenner 2010).

The mainstay treatment strategies for HNSCC include surgery, radiation therapy, and chemotherapy. Despite advancements in the therapeutic landscape, the prognosis for patients with HNSCC continues to be challenging, primarily due to delayed diagnosis, frequent recurrence at the primary site, and lymphatic metastasis. (Anderson et al. 2021). These complications have curtailed any marked improvement in long-term survival rates. While early-stage oral cavity cancers may be treated with surgery alone and laryngeal cancers with surgery or radiation, the treatment for the majority of HNSCC patients typically necessitates a combination of modalities, requiring collaborative multidisciplinary management. The overexpression of epidermal growth factor receptor (EGFR) in more than 90% of HNSCC patients led to the approval of cetuximab (Erbitux), a monoclonal antibody targeting EGFR, in combination with radiation therapy for advanced local/regional carcinoma. However, the response rate to cetuximab in HNSCC patients is less than 20% (Specenier and Vermorken 2013). Current immunotherapeutic approaches being investigated include immune checkpoint inhibitors, costimulatory agonists, vaccines targeting specific antigens, oncolytic virus therapy, adoptive T-cell transfer, and therapies targeting EGFR (Yu et al. 2022).

Recent years have seen pivotal advancements in understanding the molecular mechanisms driving HNSCC. High-throughput sequencing technologies have revealed considerable genetic heterogeneity within HNSCC tumors, identifying key alterations in tumor suppressor genes such as TP63 and oncogenes such as EGFR and PIK3CA (Alsahafi et al. 2019). TP63 is detected in approximately 80% of HNSCCs; ΔNp63 is predominantly implicated in the pathogenesis of HNSCC, modulates critical pathways such as cell survival and renewal, inhibits senescence by repressing p16/INK4A, and governs growth factor signaling (Si et al. 2016; Rocco et al. 2006). Additionally, a genomic analysis by the Cancer Genome Atlas in 2015 revealed inactivating mutations in the NOTCH1-3 gene in 17% of HPV-positive and 26% of HPV-negative HNSCC patients (Cancer Genome Atlas 2015). Moreover, PI3K/Akt/mTOR pathway disruptions are commonly observed in HNSCC, with mutations in the PIK3CA gene present in approximately 16% of cases, suggesting that this gene is a potential target for therapeutic intervention (Kang et al. 2015). Recent days, the prognostic model based on varies omics data for HNSCC were also generated. Increasing evidence has demonstrated that small nucleolar RNAs (snoRNAs) play an important role in tumorigenesis; a risk model based on SNORD114-17, SNORA36B, SNORD78, U3: ENSG00000212182, and U3: ENSG00000212195 is reported as a prognostic marker for HNSCC (Xing et al. 2020a), as well as the five-pseudogene signature (Xing et al. 2020b) (LILRP1, RP6-191P20.5, RPL29P19,TAS2R2P, and ZBTB45P1) and the six-MPS model (Xing et al. 2020c) (hsa00290, hsa01230, hsa00430, hsa00380, hsa00232, and hsa00534).

The current academic discourse around HNSCC is focused on improving the understanding of the disease pathogenesis, optimizing existing treatment protocols, and developing novel therapeutic strategies to improve patient outcomes. Aberrant accumulation of intracellular disulfides, such as cystine, induces disulfide stress and can be highly toxic to cells (Liu et al. 2020; Joly et al. 2020). Liu et al. (2023) reported that aberrant accumulation of intracellular disulfides in SLC7A11-high cells under glucose starvation induces a previously uncharacterized form of cell death distinct from apoptosis and ferroptosis, termed as disulfidptosis, which might be promoted by 90 proteins reflected by proteomic analysis. In the current study, we aim to evaluate the potential prognostic value of disulfidptosis-associated genes in HNSCC, to provide novel insights.

## Methods

### Cohort information

In this study, we collected 959 HNSCC samples from four cohorts, most of which were from the larynx, oral cavity, oropharynx, and tongue. The HNSCC cohort, comprising 509 patients, had an average age of 60.8 years, with the majority being male (74.3%). Additionally, 58.2% of the patients were alive, with an average overall survival of 30.7 months. In the EMTAB8588 cohort of 83 patients, the average age was slightly lower at 58.5 years, with a significant male majority (90.4%). A total of 63.9% of patients died, and the mean overall survival time was 58.5 months. In the GSE41613 cohort, 97 HNSCC patients, including 31 females and 66 males, met the end-of-life criteria, and the average survival time was approximately 44.1 months. Finally, the GSE65858 cohort included 270 patients, with an average age of 60.1 years and a high male predominance (82.6%). The mean overall survival was the lowest at 29.0 months, with 34.8% of the patients dying. The baseline information for patients in both cohorts is presented in Table 1.

**Table 1:** Basic information of the clinical cohorts included in the present study

	HNSCC (*N* = 509)	EMTAB8588 (*N* = 83)	GSE41613 (*N* = 97)	GSE65858 (*N* = 270)
*Age, years*				
Mean (SD)	60.8 (11.7)	58.5 (8.97)	58.9 (15.6)	60.1 (10.3)
Median [Min, Max]	61.0 [19.0, 88.0]	59.4 [26.1, 82.8]	50.5 [29.0, 74.0]	58.6 [35.3, 87.4]
*Gender*				
Female	131 (25.7%)	8 (9.6%)	31 (32.0%)	47 (17.4%)
Male	378 (74.3%)	75 (90.4%)	66 (68.0%)	223 (82.6%)
*Tumor site*				
Base of tongue	24 (4.7%)	15 (18.1%)	0 (0%)	0 (0%)
Cheek mucosa	18 (3.5%)	0 (0%)	0 (0%)	0 (0%)
Floor of mouth	52 (10.2%)	0 (0%)	0 (0%)	0 (0%)
Gum	11 (2.2%)	0 (0%)	0 (0%)	0 (0%)
Hypopharynx	9 (1.8%)	3 (3.6%)	0 (0%)	33 (12.2%)
Larynx	113 (22.2%)	20 (24.1%)	0 (0%)	48 (17.8%)
Oral cavity	24 (4.7%)	15 (18.1%)	0 (0%)	83 (30.7%)
Oropharynx	9 (1.8%)	7 (8.4%)	0 (0%)	102 (37.8%)
Others	12 (2.4%)	0 (0%)	0 (0%)	0 (0%)
Overlapping lesion of lip, oral cavity, and pharynx	68 (13.4%)	0 (0%)	0 (0%)	0 (0%)
Tongue	126 (24.8%)	0 (0%)	0 (0%)	0 (0%)
Tonsils	43 (8.4%)	23 (27.7%)	0 (0%)	0 (0%)
unknow	0 (0%)	0 (0%)	97 (100%)	4 (1.5%)
*Alcohol history*				
Yes	342 (67.2%)	51 (61.4%)	0 (0%)	239 (88.5%)
No	156 (30.6%)	31 (37.3%)	0 (0%)	31 (11.5%)
Unknow	11 (2.2%)	1 (1.2%)	97 (100%)	0 (0%)
*Smoking history*				
Yes	509 (100%)	68 (81.9%)	0 (0%)	220 (81.5%)
No	0 (0%)	14 (16.9%)	0 (0%)	48 (17.8%)
Unknow	0 (0%)	1 (1.2%)	97 (100%)	2 (0.7%)
*Status*				
Alive	296 (58.2%)	30 (36.1%)	46 (47.4%)	176 (65.2%)
Dead	213 (41.8%)	53 (63.9%)	51 (52.6%)	94 (34.8%)
*Over survival, months*				
Mean (SD)	30.7 (28.5)	58.5 (44.3)	44.1 (26.5)	29.0 (14.8)
Median [Min, Max]	21.8 [0.980, 210]	47.0 [0.130, 139]	54.4 [0.460, 85.0]	27.4 [0.360, 78.5]

### Mitigating batch effects

Batch effects represent the nonbiological discrepancies observed across multiple datasets. To ensure analytical consistency and mitigate biases introduced by such effects, we employed the ComBat algorithms from the "sva" package. This methodology was instrumental in harmonizing the transcriptional profiles of all the enrolled cohorts, thus effectively offsetting the intrinsic batch differences among them. Subsequently, all four cohorts were used for consensus clustering, and in the prediction of the prognostic model, the training cohort, called Cohort A, was combined with the EMTAB8588, GSE41613 and GSE65858 cohorts, while the TCGA-HNSCC cohort was used as validation Cohort B.

### Comparison of genes associated with disulfidptosis

Liu and colleagues delineated a novel classification system for disulfidptosis. They observed that cells with elevated levels of SLC7A11, when exposed to glucose deficiency, experienced increased uptake of cystine. This surge, concomitant with an insufficient supply of NADPH, precipitates the exhaustion of NADPH stores, leading to improper disulfide bond formation in actin cytoskeletal proteins, disassembly of the actin network, and subsequent cellular collapse (Liu et al. 2023). A total of 90 cysteine residues exhibited a 1.5-fold increase in disulfide bonding after the cells were subjected to glucose deficit. These residues were encoded by 77 distinct genes, with their identifiers collated from Supplementary Table 2 in the study by Liu et al.

### Elucidating distinct disulfidptosis phenotypes

To discern molecular subtypes, consensus clustering was performed using the "ConsensusClusterPlus" package in R (Wilkerson and Hayes 2010). This process involved 50 iterations of k-means clustering, with each iteration incorporating at least 80% of the sample set. The cumulative distribution function (CDF) score—representative of the area beneath the CDF curve—was computed to ascertain the optimal number of clusters. Both principal component analysis (PCA) and t-distributed stochastic neighbor embedding (t-SNE) were utilized to validate the integrity of the consensus clusters.

### Gene set enrichment analysis

The identification of differentially expressed genes (DEGs) is pivotal for revealing distinct mechanistic pathways across subgroups. The 'limma' package in R facilitated the compilation of DEGs, setting an adjusted *P* value of less than 0.01 as the threshold for selection. Gene Ontology (GO), Kyoto Encyclopedia of Genes and Genomes (KEGG), and HALLMARK enrichment analyses were conducted using the 'org.Hs.eg.db' and 'msigdbr' packages (Ashburner et al. 2000; Liberzon et al. 2015), while 'ClusterProfiler' elucidated key signaling pathways (Yu et al. 2012).

### Formulating the prognostic index

Prognostic genes were initially identified through univariate Cox regression in Cohorts A and B. A Venn diagram was then constructed to highlight the prognostic genes across both cohorts for further examination. The 'glmnet' package in R facilitated the implementation of LASSO regression analysis to distill and regularize input variables, thereby deriving a prognostic model of superior predictive power and interpretability (Friedman et al. 2010). LASSO is a regression analysis method that performs both variable selection and regularization to enhance the prediction accuracy and interpretability of the resulting statistical model. Genes identified through LASSO were instrumental in computing each patient's risk score, and the disulfidptosis prognostic signature (DSPS) was established through the aggregation of gene expression levels and corresponding coefficients, where DSPS = sum (gene expression × corresponding coefficient).

### Construction of a predictive nomogram

Comprehensive multivariate Cox regression analysis revealed the preliminary phase in identifying independent prognostic factors. Following this, the DSPR signature was incorporated into the model. A nomogram illustrating the independent prognostic factors to consolidate the forecasted outcomes was subsequently developed using the "rms" and "regplot" R packages. Calibration plots, decision curve analysis (DCA), and clinical impact curve assessments substantiated the precision and dependability of our prognostic data.

### Personalized chemotherapy prediction

The "pRRophetic" R package predicted individual chemotherapeutic responses by harnessing drug sensitivity and phenotypic data from GDSC 2016 (Geeleher et al. 2014a). Ridge regression yielded estimated IC50 values for each specimen in response to specific chemotherapeutic agents, with lower IC50 values indicating greater susceptibility to treatment (Arthur 2000), while the prediction accuracy was verified through a tenfold cross-validation method (Geeleher et al. 2014b).

### Statistical analysis

The *t* test is a parametric test used for comparing the means of two groups, assuming a normal distribution and equal variances. Moreover, the Mann‒Whitney test is a nonparametric test used to compare the distributions of two groups without assuming a specific data distribution and is more robust to outliers. K‒M survival analysis and log-rank tests were used to generate survival curves, while univariate Cox regression models were used to calculate hazard ratios (HRs) and 95% confidence intervals (CIs) for selected genes. Multivariate Cox regression confirmed the independent prognostic significance of the risk score after adjusting for various clinical parameters. Statistical significance was determined using a two-tailed *P* value of less than 0.05, with all the statistical analyses performed in R software (version 4.2.2).

## Results

### Unveiling subclasses within the definition of disulfidptosis

**Fig. 1:**
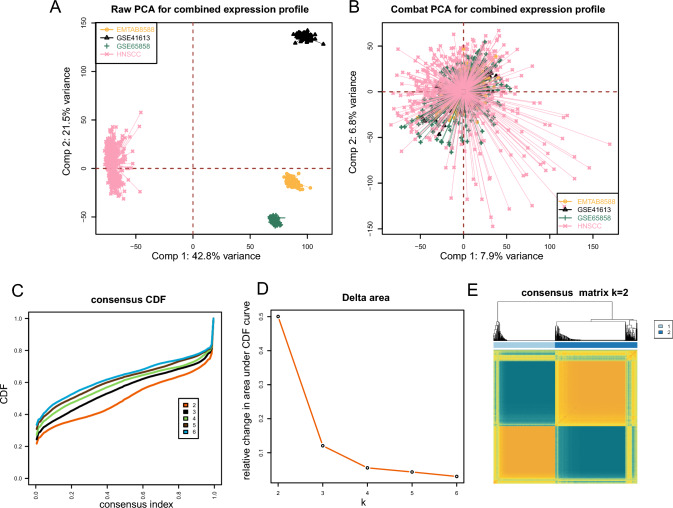
Batch correction and consensus clustering in HNSCC cohorts. **A** Raw distribution of gene expression of samples in the enrolled cohorts before batch correction. **B** Distribution of samples in the combined expression profile after batch correction. **C** Consensus cumulative distribution function (CDF) for *k* = 2 to 6 clusters. **D** Delta area under the CDF curve for different *k* values. **E** Consensus matrix for *k* = 2 indicating two distinct clusters

Our study included 959 HNSCC patient samples from four cohorts, as outlined in “Methods”. To ensure the consistency of the transcriptomic data prior to further analysis, we initially performed batch correction on the gene expression profiles from all four cohorts. Prior to this correction, principal component analysis (PCA) graphs exhibited notable differences between the four cohorts (Fig. 1A). However, postcorrection, batch effects on gene expression distribution across all cohorts were effectively mitigated (Fig. 1B).

As detailed in “Methods”, we first collated 79 disulfidptosis-related genes, 68 of which were uniformly distributed across the included cohorts. Hence, the gene expression profiles of these 68 genes were gathered to serve as the input matrix for clustering. Consensus matrices for k values from 2 to 6 were tested (Fig. 1C). The cumulative distribution function (CDF) curves revealed that the area under the CDF did not change drastically with the increase in the number of k after 2 (Fig. 1D), and the relative change in the CDF was maximal between *k* = 2 and *k* = 3. Therefore, we determined that *k* = 2 was the optimal number of clusters for subsequent analysis (Fig. 1E).

**Fig. 2:**
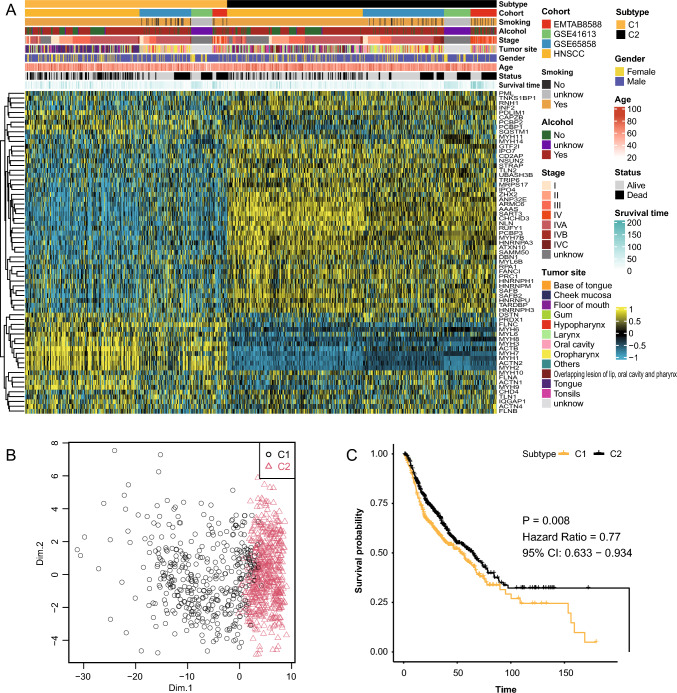
Subclassification of HNSCC based on disulfidptosis-related genes. **A** Heatmap displaying the expression profiles of 68 genes in the two subgroups. **B** The results of random forest showing the separation of the C1 and C2 subgroups. **C** Kaplan‒Meier survival curves comparing the prognosis of patients in subgroups C1 and C2

Thus, the 959 patients from all cohorts were classified into two subgroups, C1 and C2, based on the expression of 68 genes (Fig. 2A). Patients in subgroup C1 exhibited increased expression of genes from the MYH family, ACTB, ACTN2, and FLNC, whereas patients in subgroup C2 displayed increased expression of other genes, particularly AAAS, ARMC6, SART3, and CHCHD3. A random forest classification based on the expression profiles of the 68 genes also confirmed the distinction between the two groups (Fig. 2B). The prognosis of patients from different subtypes was examined. There were 411 individuals in subtype C1, with 199 deaths, representing a mortality rate of 48.4%; subtype C2 comprised 548 individuals, with 212 deaths, representing a mortality rate of 38.7%, indicating a better prognosis for patients in subtype C2 (HR = 0.77, 95% CI: 0.633–0.934, *P* = 0.008; Fig. 2C).

### Key genes and pathways impacted by disulfidptosis

**Fig. 3:**
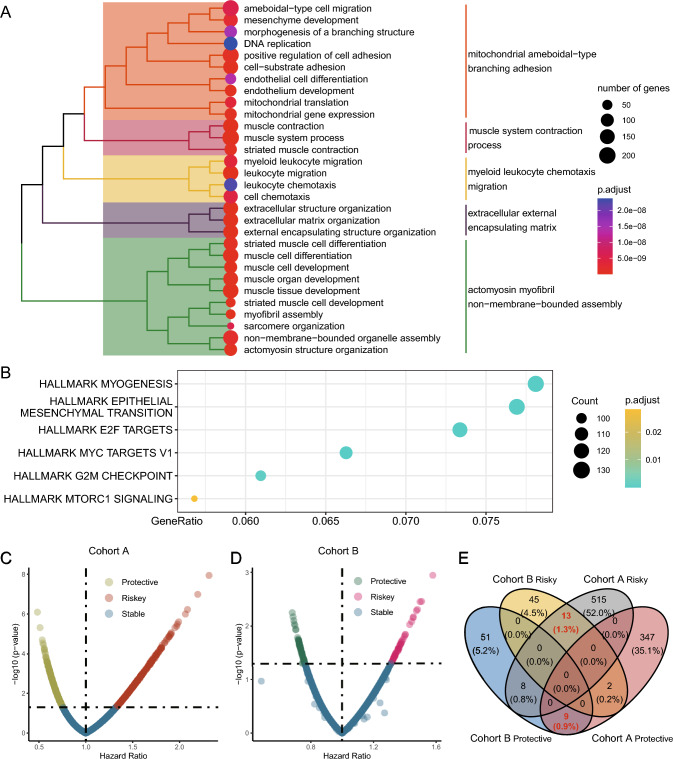
Pathway enrichment analysis and prognostic gene isolation. **A** Gene Ontology term enrichment analysis for identified differentially expressed genes. **B** HALLMARK pathway enrichment analysis highlighting key biological processes. **C** Volcano plot of prognostic genes in Cohort A. **D** Volcano plot of prognostic genes in Cohort B. **E** Venn diagram showing overlap of risk-related and protective genes across cohorts

Due to the significant differences in OS between subtypes C1 and C2, we compared the DEGs between these subtypes. Using a *P* value threshold of less than 0.05, we identified 4943 DEGs. Moreover, to investigate the potential functional mechanisms of these genes, we conducted pathway enrichment analyses, which suggested that disulfidptosis may affect signaling pathways related to extracellular matrix structure, cellular development and differentiation, cell adhesion, myeloid leukocyte chemotaxis, migration, and mitochondrial function (Fig. 3A). Additionally, HALLMARK terms indicated that these genes were involved in signaling pathways related to epithelial–mesenchymal transition, cell cycle-associated E2F targets, MYC targets, and the G2M checkpoint (Fig. 3B).

### Disulfidptosis-related genes associated with patient prognosis

We assessed the prognostic significance of these 4943 genes in Cohort A and Cohort B. In Cohort A, we identified 659 risk-related genes (HR > 1, *P* < 0.05) and 468 protective genes (HR < 1, *P* < 0.05) (Fig. 3C), and in Cohort B, we found 98 risk-related genes (HR > 1, *P* < 0.05) and 87 protective genes (HR < 1, *P* < 0.05) (Fig. 3D). After filtering for genes that exhibited similar trends in both cohorts, we identified 13 risk-related genes and 9 protective genes associated with the prognosis of HNSCC patients (Fig. 3E).

### Prognostic model based on disulfidptosis-related genes

**Fig. 4:**
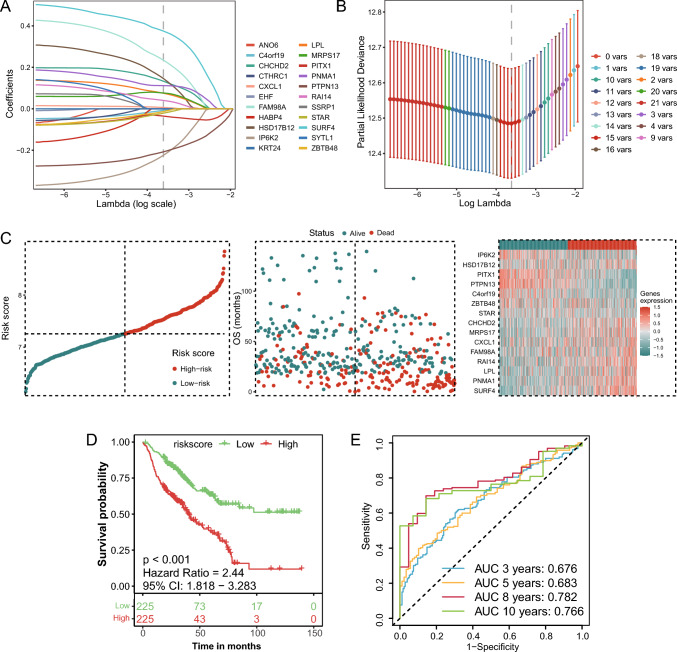
Development of the disulfidptosis prognostic signature (DSPS). **A** LASSO coefficient profiles of the disulfidptosis-related genes. **B** Selection of the optimal lambda value in the LASSO model. **C** Risk score distribution, patient status, and gene expression heatmap for the DSPS model. **D** Kaplan‒Meier curves for the high- and low-DSPS groups. **E** ROC curves evaluating the prognostic accuracy of the DSPS

We included the 22 genes identified via LASSO regression to further filter HNSCC prognosis-related genes and establish a prognostic prediction signature. In the LASSO pathway diagram (Fig. 4A), each line represents a gene in the model. With the smallest lambda value at 0.0269, the partial likelihood deviation also met the minimum, reflecting the optimal formula and preventing overfitting (Fig. 4B). Ultimately, the LASSO regression model selected 15 out of the 22 input genes, constructing a prognostic feature known as the DSPS. DSPS = (MRPS17 × 0.076) + (LPL × 0.075) + (CHCHD2 × 0.133) + (FAM98A × 0.235) + (CXCL1 × 0.006) + (SURF4 × 0.375) + (PNMA1 × 0.112) + (RAI14 × 0.04) + (HSD17B12 × 0.146) + (C4orf19 × − 0.007) + (IP6K2 × − 0.224) + (PTPN13 × − 0.208) + (STAR × − 0.021) + (PITX1 × − 0.035) + (ZBTB48 × − 0.013). Subsequently, we calculated a risk score for each patient in Cohort A based on the DSPS, with the dot plot on the left displaying each patient's calculated DSPS, the scatter plot in the middle depicting each patient's status, and the heatmap on the right showing the expression of the 15 genes between the two groups (Fig. 4C).

Patients were then divided into low- and high-score groups, with those in the high-DSPS group exhibiting poorer OS than those in the low-DSPS group, with a hazard ratio of 2.44 and 95% confidence interval ranging from 1.818 to 3.283 (Fig. 4D). The ROC curve was then used to assess the predictive value of the overall clinical outcomes, showing 3-year AUCs of 0.676, 5-year AUCs of 0.683, 8-year AUCs of 0.782, and 10-year AUCs of 0.766 (Fig. 4E). After excluding the potential influences of other clinical parameters (such as patient age, sex, primary tumor location, tumor stage, smoking history, and alcohol consumption history), the DSPS was determined to be an independent prognostic indicator (HR = 2.055, 95% CI: 1.420–2.975, *P* < 0.001), as was tumor stage (stage IVB: HR = 3.13, 95% CI: 1.196–8.193, *P* = 0.02; stage IVC: HR = 2.785, 95% CI: 1.001–7.748, *P* = 0.05; Table 2).

**Table 2:** Results of multivariate Cox regression analysis in the training cohort

Parameters	Subcategory	HR	95% CI	*p* Value
Age		1.03	1.011–1.049	0.002*
Gender	Female	Ref.		
	Male	0.952	0.587–1.544	0.842
Stage	I	Ref.		
	II	0.472	0.194–1.151	0.099
	III	0.807	0.374–1.744	0.586
	IV	0.936	0.417–2.102	0.873
	IVA	1.171	0.581–2.361	0.66
	IVB	3.13	1.196–8.193	0.02*
	IVC	2.785	1.001–7.748	0.05*
Tumor site	Base of tongue	Ref.		
	Hypopharynx	1.822	0.755–4.394	0.182
	Larynx	1.178	0.53–2.618	0.688
	Oral cavity	1.157	0.503–2.661	0.732
	Oropharynx	0.864	0.376–1.986	0.731
	Tonsils	0.708	0.288–1.739	0.451
Alcohol history	No	Ref.		
	Yes	1.158	0.715–1.877	0.551
Smoking history	No	Ref.		
	Yes	1.186	0.736–1.913	0.483
Risk	Low DSPS	Ref.		
	High DSPS	2.055	1.42–2.975	1.34E−04*

^*^P < 0.05

### Molecular mechanisms and suitable chemotherapeutic drugs for the DSPS subgroups

**Fig. 5:**
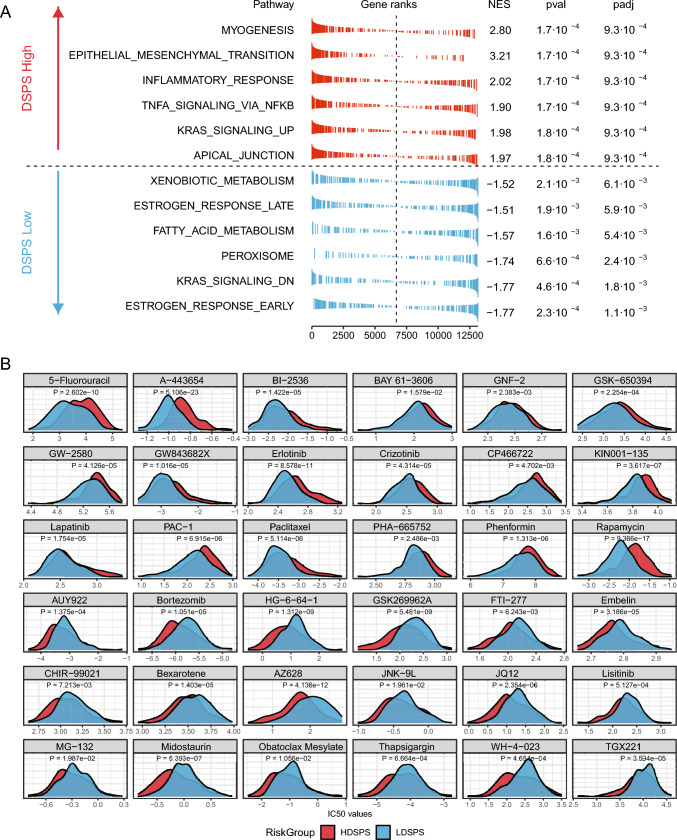
Molecular mechanisms and drug susceptibility of the DSPS subgroups. **A** Pathway activation levels in the high- vs. low-DSPS subgroups. **B** Differential drug sensitivity analysis between the DSPS subgroups

To understand the underlying molecular characteristics of patients divided into high-DSPS and low-DSPS subgroups, or to explore the molecular mechanisms underlying the different clinical outcomes of the two groups, we evaluated the activation levels of 50 cancer-related pathways from the HALLMARK cohort to assess the unique molecular mechanism features of the two subgroups. We observed that in the high-DSPS subgroup, pathways related to epithelial–mesenchymal transition, apical junction, and immune-related pathways exhibited increased activation, while in the low-DSPS subgroup, pathways related to estrogen response, fatty acid metabolism, and KRAS signaling exhibited increased activation (Fig. 5A).

Further studies aimed at guiding clinical treatment for HNSCC utilized the "pRRophetic" R package to analyze the differences in the efficacy of 86 potential chemotherapeutic drugs between the different subgroups. Patients in the low-DSPS group benefited more from treatment with drugs such as 5-fluorouracil, A-443654, BI-2536, BAY 61-3606, GNF-2, GSK-650394, GW-2580, GW843682X, erlotinib, crizotinib, CP466722, KIN001-135, lapatinib, PAC-1, paclitaxel, PHA-665752, phenformin, and rapamycin, while patients with a high-DSPS score might benefit more from treatment with AUY922, bortezomib, HG-6-64-1, GSK269962A, FTI-277, embelin, CHIR-99021, bexarotene, AZ628, JNK-9 L, JQ12, linsitinib, MG-132, midostaurin, Obatoclax mesylate, thapsigargin, WH-4-023, and TGX221 (all *P* < 0.05, Fig. 5B).

### Clinical characteristics and the DSPS used to construct the prognostic nomogram

**Fig. 6:**
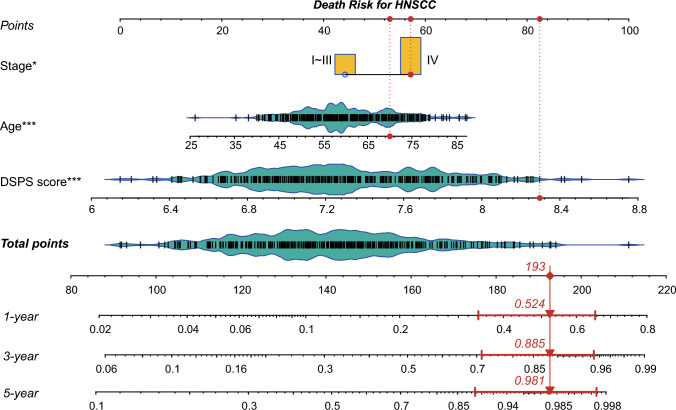
Prognostic prediction nomogram incorporating the clinical characteristics and the DSPS

Multivariate Cox regression analysis revealed that age, tumor stage, and the DSPS are independent factors for the prognosis of HNSCC patients. Hence, we constructed a prognostic prediction nomogram based on these three indicators (Fig. 6). For instance, as marked with red lines and dots, a 70-year-old male HNSCC patient with a postoperative tumor stage of IV and a DSPS of 8.3 had a cumulative score of 193, corresponding to a 1-year mortality risk of 0.524, a 3-year mortality risk of 0.885, and a 5-year mortality risk of 0.981 (Fig. 6).

**Fig. 7:**
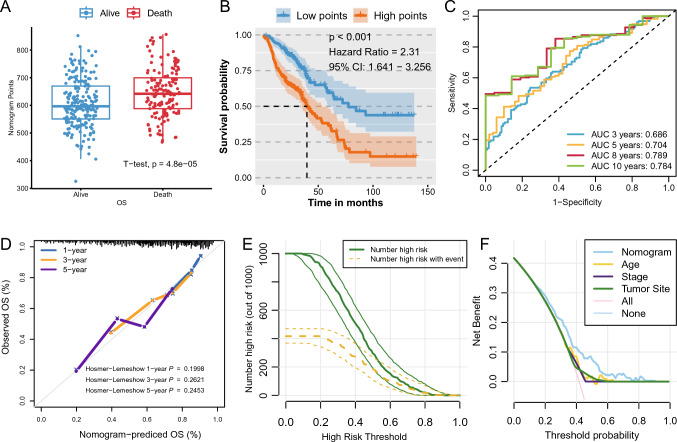
Validation and clinical utility of the prognostic nomogram. **A** Boxplot showing the nomogram point distribution by survival status. **B** Kaplan‒Meier curves for risk stratification based on nomogram points. **C** ROC curve analysis of the nomogram's prediction accuracy. **D** Calibration curves for the nomogram at 1, 3, and 5 years. **E** Clinical impact curves demonstrating the benefit of nomogram-based risk stratification. **F** DCA showing the net benefit of the nomogram across different risk thresholds

Based on the aforementioned nomogram, we calculated points for all patients and found that those who died had significantly more points than those who still survived at the last follow-up (*P* < 0.01, Fig. 7A). Dividing the patients according to the median points into high and low groups revealed that patients with higher points had a worse prognosis (HR = 2.31, 95% CI: 1.641–3.256, *P* < 0.001; Fig. 7B). The predictive accuracy of the nomogram was further improved, with 3-year AUC values of 0.686,5-year AUC values of 0.704, and 8-year AUC values of 0.789 (Fig. 7C). Calibration curves also indicated a high consistency between the nomogram's prognostic prediction and the actual outcomes, with Hosmer–Lemeshow *P* values at 1 year, 3 years, and 5 years of 0.199, 0.262, and 0.245, respectively, confirming the nomogram's commendable ability to predict clinical outcomes (Fig. 7D). Clinical impact curves suggested that treating all patients with a predicted clinical death probability greater than 70% could yield the most significant clinical benefit (Fig. 7E). Decision curve analysis (DCA) demonstrated that using the DSPS feature to predict the risk of death provided an equal or greater benefit than treating all or no patients when the threshold probability exceeded 20% (Fig. 7F).

### The DSPS successfully identified high-risk patients in the validation cohort

**Fig. 8:**
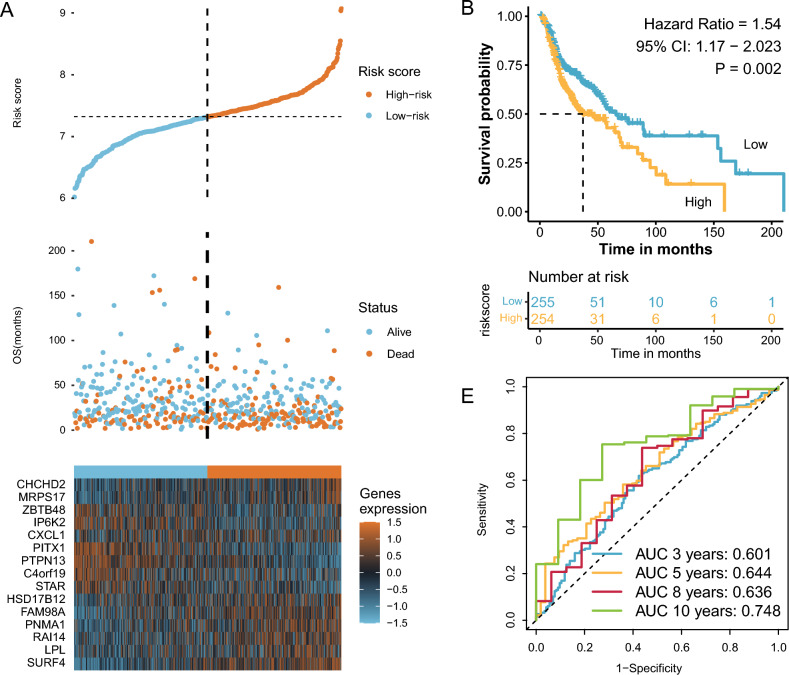
Validation of the DSPS in an external cohort. **A** Distribution of risk scores and survival status in the validation cohort. **B** Kaplan‒Meier curves for the high- and low-DSPS groups in the validation cohort. **C** ROC curves evaluating the prognostic accuracy of the DSPS

Using the DSPS formula derived from the LASSO analysis based on Cohort A, we calculated the risk score for each patient in the validation cohort, Cohort B (Fig. 8A). Patients were divided into low and high-DSPS groups, with the high-DSPS group showing worse overall survival outcomes than the low-DSPS group (HR = 1.54, 95% CI: 1.17–2.023; *P* = 0.002; Fig. 8B). Furthermore, the AUC values demonstrated satisfactory results, with 3-year survival at 0.601, 5-year survival at 0.644, 8-year survival at 0.636, and 10-year survival at 0.748 (Fig. 8C). After conducting multifactorial Cox regression analysis to eliminate the impact of other factors on prognosis, after adjusting for factors such as sex, age, tumor stage, and history of alcohol consumption, the DSPS could serve as an independent prognostic indicator (HR = 1.542, 95% CI: 1.137–2.091, *P* = 0.005; Table 3).

**Table 3:** Results of multivariate Cox regression analysis in the validation cohort

Parameters	Subcategory	HR	95% CI	*p* Value
Age		1.024	1.008–1.04	0.003*
Gender	Female	Ref.		
	Male	0.942	0.655–1.356	0.748
Stage	I	Ref.		
	II	2.079	0.712–6.068	0.181
	III	2.747	0.95–7.944	0.062
	IVA	3.99	1.45–10.983	0.007*
	IVB	8.749	2.547–30.057	5.72E−03*
Tumor site	Base of tongue	Ref.		
	Cheek mucosa	0.732	0.222–2.418	0.609
	Floor of mouth	1.427	0.54–3.773	0.474
	Gum	0.722	0.205–2.544	0.612
	Hypopharynx	1.1	0.258–4.695	0.898
	Larynx	0.64	0.246–1.666	0.36
	Oral cavity	0.782	0.234–2.62	0.691
	Oropharynx	0.776	0.086–7.042	0.822
	Others	0.585	0.136–2.512	0.471
	Overlapping lesion of lip, oral cavity, and pharynx	1.045	0.399–2.736	0.929
	Tongue	1.14	0.448–2.903	0.783
	Tonsils	0.554	0.157–1.951	0.358
Alcohol history	No	Ref.		
	Yes	1.071	0.766–1.498	0.689
Risk	Low DSPS	Ref.		
	High DSPS	1.542	1.137–2.091	0.005*

^*^P < 0.05

## Discussion

The notion of disulfidptosis and its link to HNSCC prognosis has been substantiated by our extensive cohort analysis, underscoring the use of the DSPS as a robust prognostic indicator. This study, encompassing diverse patient datasets, has meticulously accounted for batch effects, thus bolstering the integrity of the gene expression data that underpins our signature. We firstly identified two disulfidptosis subtypes by consensus clustering, and then 4943 differentially expressed genes between the above two subtypes were selected and their prognostic value evaluated in two independent clinical cohorts. Thirdly, 13 risk-related genes and 9 protective genes associated with the prognosis of HNSCC patients were chosen. Finally, the prognostic DPSP signature was generated by the LASSO regression analysis.

Our findings indicate that subtype C2, characterized by lower mortality rates, may represent a less aggressive form of HNSCC. In contrast, the higher mortality of subtype C1 could indicate a more invasive disease trajectory and greater expression of genes from the MYH family, ACTB, ACTN2, and FLNC. Several studies have reported the prognostic value of the MYH family genes MYL1, MYL2, MYH1, MYH2, and MYH7, which are unfavorable prognostic markers in HNSCC and might promote CD4 + T-cell activation (Li et al. 2023; Ju et al. 2023). The level of phosphorylated MYH2-Y1381 is reportedly significantly lower in recurrent HNSCC patients than in primary patients (Kaneko et al. 2022). Hub genes might be pivotal components that accelerate the progression of HNSCC. Zhang et al. (2018) identified ten hub genes by weighted gene coexpression network analysis, indicating the influence of MMP1, TNFRSF12A, PLAU, FSCN1, PDPN, KRT78, EVPL, GGT6, SMIM5, and CYSRT1. Focusing on the hub genes might support molecular targeted therapeutic drug development.

The constructed DSPS, derived from LASSO regression analysis, encapsulates the prognostic importance of disulfidptosis-related genes, including MRPS17, LPL, CHCHD2, FAM98A, CXCL1, SURF4, PNMA1, RAI14, HSD17B12, C4orf19, IP6K2, PTPN13, STAR, PITX1, and ZBTB48. CXCL1 plays a role in the etiology of HNSCC, with its overexpression observed in fibroblasts within oral submucous fibrosis—a precancerous condition. This chemokine facilitates oncogenic activities in oral submucous fibrosis by inducing keratinocyte proliferation and migration and enhancing the stem-like properties of these cells (Ye et al. 2018). FAM98A was shown to promote the progression of endometrial carcinoma (Li et al. 2019), non-small cell lung cancer (Zheng et al. 2018), and breast cancer (Liu et al. 2021), and we also revealed the role of FAM98A in the risk of HNSCC in the present study. SURF4 interacts with the ERGIC53 and p25 proteins and engages with STIM1 within the endoplasmic reticulum (ER) lumens. It plays a regulatory role in STIM1-mediated store-operated calcium entry (SOCE), a fundamental mechanism for calcium influx in cells (Fujii et al. 2012). In tumors, SURF4 reportedly promotes tumorigenesis in breast cancer (Zhai et al. 2022), ovarian cancer (Yue et al. 2020), and myeloid leukemia (Kim et al. 2023).

Furthermore, the DSPS has proven to be an independent prognostic factor, suggesting that it could be used alongside traditional staging systems to more accurately predict patient outcomes. The construction of a prognostic nomogram that integrates the DSPS with clinical characteristics such as age and tumor stage represents a significant step toward personalized medicine. The nomogram's predictive accuracy, as indicated by the area under the curve (AUC) values and calibration curves, enhances its clinical utility, potentially aiding clinicians in making informed decisions about patient management.

The molecular pathways associated with disulfidptosis revealed in our study shed light on potential mechanisms that could drive the observed prognostic differences. For instance, the activation of pathways related to epithelial–mesenchymal transition and immune response in the high-DSPS subgroup points to a more aggressive disease phenotype, which is corroborated by their poorer prognosis. These pathways may serve as targets for novel therapies that could mitigate the aggressive nature of HNSCC in these patients. Moreover, our study's personalized chemotherapy prediction suggested that the DSPS can inform treatment choices. Patients in the low-DSPS group could benefit from a range of drugs, including well-established agents such as 5-fluorouracil, indicating the potential for individualized treatment plans based on the molecular profile of the tumor.

Our study, however, is not without limitations. Although the DSPS holds promise, its application in clinical practice requires further validation in prospective studies. The cohorts used in this study, although diverse, may not capture all the nuances of the global HNSCC population. Additionally, the mechanisms underlying disulfidptosis and its impact on HNSCC progression need to be elucidated through functional studies.

## Conclusion

In summary, the DSPS has emerged as a potent prognostic tool that captures the complexity of HNSCC. This study provides insight into the future of oncology, where molecular signatures could guide therapeutic decisions and improve patient outcomes. As we move toward a more tailored approach to cancer treatment, the DSPS stands as a testament to the power of genomic medicine in improving the prognosis and management of HNSCC.

## Data Availability

The datasets analyzed during the current study are available from the corresponding author upon reasonable request.
